# Ecology of War, Health Research and Knowledge Subjugation: Insights from the Middle East and North Africa Region

**DOI:** 10.5334/aogh.3015

**Published:** 2020-09-18

**Authors:** Nassim El Achi, Marilyne Menassa, Richard Sullivan, Preeti Patel, Rita Giacaman, Ghassan S. Abu-Sittah, on behalf of R4HC-MENA

**Affiliations:** 1 Conflict Medicine Program, Global Health Institute, American University of Beirut, Beirut 1107 2020, LB; 2 Institute of Cancer Policy, Cancer Epidemiology, Population & Global Health, School of Cancer Sciences, King’s College London, London, SE1 9RT, UK; 3 Conflict and Health Research Group, King’s College London, London, UK; 4 Department of War Studies, King’s College London, London WC2R 2LS, UK; 5 Institute of Community and Public Health, Birzeit University, Birzeit, West Bank, PS

## Abstract

In an ecology of war, as experienced in the Middle East and North Africa region, health research faces several interrelated challenges: de-prioritization, paucity in the generation of reliable data, and its securitization. This directly contributes to local knowledge subjugation and research waste as local narratives are disqualified in favor of institutionalized and privileged global unitary knowledge. Huge efforts that require political will and commitment, coupled with multidisciplinary approaches and sustainable collaborations between researchers and humanitarian workers at the local, regional and global levels, are indispensable to give more space for the abandoned local knowledge in order to have contextualized and more impactful interventions where more lives are saved.

## Introduction

The Middle East and North Africa (MENA) region is plagued with protracted armed conflicts along with political unrest that has resulted in some of the worst humanitarian crises and the greatest waves of forced migration since World War II. For instance, 37% of 70.8 million people displaced worldwide as of 2018 originate from the MENA region [1]. This reality alters the region’s living environment creating an ecology of war that continues far beyond the ceasefire. The home becomes the new battlefront, with continuing physical, and often invisible psychological and social wounds [2]. In such a *de facto* ecology of war, Foucault’s *subjugated knowledge* becomes inevitable where certain discourses are disqualified in favor of the institutionalized and privileged “global unitary knowledge,” which are considered the legitimate and formal knowledge domains [3]. Although knowledge subjugation has been a topic of concern among various fields including terrorism, aboriginal and postcolonial studies, such a focus is not found in the literature that tackles health research in fragile and protracted conflict settings such as the MENA region [4,5,6] (see **Box 1** for definition).

**Box 1: Key definitions.**

***The MENA (Middle East and North Africa) Region:*** Covers 24 countries, namely the 21 members of the Arab League (Algeria, Bahrain, Djibouti, Egypt, Iraq, Jordan, Kuwait, Lebanon, Libya, Mauritania, Morocco, Oman, Palestine, Qatar, Saudi Arabia, Somalia, Sudan, Syria, Tunisia, the United Arab Emirates, and Yemen), and could also include Iran, Israel and Turkey [9].

***Protracted Conflict:*** Hostile interactions that extend over long periods of time with sporadic outbreaks of open warfare fluctuating in frequency and intensity [10]. In the MENA region, interstate armed conflicts are on average of 14 months duration, and intrastate armed conflicts are on average of four years duration, and counting…[11]

***Ecology of war:*** is the result of protracted conflicts which maintain themselves well beyond the end of shooting, and alter the living environment of people who struggle with physical, psychological and social wounds; “… with the deliberate destruction of health provision, and the repeating cycles of infection, injury, poverty, and human misery, all of which become a permanent reality [12]”

***Subjugated knowledge:*** “…historical contents that have been buried and disguised in a functional coherence or formal systemization. […] a whole set of knowledge that has been disqualified as inadequate to the task or insufficiently elaborated [3]”

As a continuation of our work on the ecology of war at the Conflict Medicine Program at the Global Health Institute at the American University of Beirut, and as part of the Research for Health in Conflict MENA project (R4HC-MENA) where we seek to provide contextualized and multidisciplinary knowledge about contemporary health issues in conflict settings, we here discuss three tightly linked reasons that are driving local knowledge subjugation in conflict [7,8]. These reasons are de-prioritization of health research, paucity in the generation of reliable data, and securitization of the data generated in conflict settings. They are part of the region’s ecology of war and form a negative cycle between health research and local knowledge subjugation.

## Why health research and local knowledge are subjugated in the MENA region

Firstly, research including health research is deprioritized in the MENA region compared to other fields such as defense, public spending, and infrastructure, and thus faces serious challenges such as the lack of a research culture, very limited local and regional funding schemes, generally weak institutional incentives for research and political sensitivity of health research findings. Consequently, the MENA region contributes to about 1.5% of research production on the global scale, dominated by few academic and research institutes in the region, with research and development expenditures averaging around 0.93% of GDP [13,14]. Nonetheless, the translation of the *limited* knowledge produced by research into health policy is frequently hampered and thus barely used in the decision-making process. The usual assumption is that policymakers rely on evidence when setting their agendas. However, policymaking in the region takes place in a political context, where conflict and power are operational in identifying priorities which may or may not be related to the availability of evidence [14].

Even within the health research discourse, there is an additional layer of knowledge subjugation as certain health research topics are emphasized by virtue of external funding research agendas and dominant Western ethno-linguistic perspectives while disregarding most of the time, local knowledge and narratives that would result in more contextually relevant research. For instance, topics such as research ethics and social wounds that are socio-culturally relevant are heavily under-researched, with most studies focusing on epidemiology, clinical trials, and biomedical research [15]. Furthermore, recent literature reviews on health research also confirm the presence of significant research gaps: A mismatch between published research and the burden of diseases in the conflict-affected MENA countries, emphasizing the need to fund and produce quality evidence from local narratives that inform reliable health policies [16].

Secondly, the shortage in the availability of reliable data within this ecology of war also contributes significantly to knowledge subjugation. For instance, data generation is constantly challenged by the prevailing political climate, logistics, safety of both participants and researchers, and limited access to archives (if existent), all of which impede the production of representative data and narratives which would help in resurfacing local subjugated knowledge. If available, data will likely be fragmented, not easily updated, nor analyzed. Despite the increase in research papers that are led by local researchers, reports on health data from Iraq and Syria highlight the difficulty in identifying the transparency of the data collected and the lack of access to national datasets [17]. While this may also be relevant to other conflict-affected regions, the intensified warfare and the breakdown of the Syrian Government services obliged humanitarian organizations to establish data collection systems to provide essential services in contested areas [18].

Thirdly, according to securitization theory, an issue is securitized when a political actor defines or articulates it as a matter of national security, which justifies the use of extraordinary measures often involving the security sector (military, police, etc.) [19]. Indeed, political and armed groups consider data regarding population figures, mortality, and spread of diseases as highly politicized or threatening to national security and thus are securitized and subject to destruction or concealment [20]. In the MENA region, there are regulations and laws that prohibit and penalize the collection and dissemination of information without government approval, but to different degrees across the region’s countries [21]. Researchers in the MENA region are sometimes subjected to political pressure and censorship practices, particularly when universities and research institutions are under the direct political influence of governments [22]. Moreover, in certain MENA countries, reporters have been subject to expulsion, suspension, or detention when criticizing governmental responses to the COVID-19 pandemic or for claiming that these governments have purposively under-reported the numbers of infections [23]. Reports generated from international development agencies such as the United Nations (UN), World Bank, and the World Health Organization (WHO) depend to a large extent on data gathered through local governmental surveillance and statistics departments. Given securitization of data, studies highlight the need to treat data provided by governments with caution and to consider them as (crude) estimates rather than actual figures [24]. Indeed a report published by the World Bank highlighted that the MENA region is the only region whose data capacity and transparency have dropped remarkably between 2005 and 2018 [25]. This could be attributed to pressure on governments to securitize data following the Arab Spring and other armed conflicts resulting in humanitarian crises. As for ongoing armed conflicts, the problem is exacerbated. Reports highlight the difficulty in accessing and disseminating data and other health-related information were fighting groups tend to use the data available to serve their purposes, as it is the case in Yemen [26].

## What can be done beyond research capacity strengthening?

With the rise in new forms of conflict around the globe, ecologies of war have become a pervasive reality. This reality has prompted the MENA-based researchers to realize that, despite all of the challenges, it is crucial to de-subjugate local knowledge and provide new approaches for health research in conflict. Local idioms and “invisible wounds” that are the result of the ecology of war are currently being included in health research to some extent in order to produce an alternative understanding of reality [15]. Indeed without opening up to new possibilities and generating new forms of knowledge, health research in the region will never be able to provide reliable data that leads to significant evidence-based and contextualized interventions that have a powerful social and political impact.

Following Pierre Bourdieu’s “collective intellectual,” it is also crucial to bring together different regional insights from various disciplines to form “critical networks” to better understand the region’s war ecology [4]. Effective mechanisms for the transfer of knowledge created by health research to the health professionals’ education systems will be able to produce a new generation of healthcare professionals who are better equipped to deal with the specificities of the region’s health system [12].

At the same time, the protracted nature of the conflict makes both the humanitarian system, with its short-term health objectives, and its own unique set of socially, economically and politically driven agendas, and the disconnected academic research no longer fit for regional needs [27]. This speaks to the need for a new research ‘contract’ between humanitarian actors and academic researchers, both local and international, to tackle the challenge of data transparency, availability, and reliability in conflict by using advanced technology such as e-health and m-health [28,29]. Moreover, decentralization of data generation and research, accompanied with a high level of coordination, can result in less biased data and more accountability to inform decision-making and foreign policy to better forecast the impact of future conflicts on health as well as to improve resilience.

The global health community, including international health institutions, have begun investing efforts in collaboration with local stakeholders and scholars to improve the capacity of health research in the region by using multidisciplinary approaches, tapping into different research fields, giving more space to previously abandoned local narratives and knowledge and sponsoring more local partnerships and networks [7]. Such efforts also serve to strengthen the capacity of the international stakeholders to better understand, analyze and interpret findings in line with the region’s contextual reality. In other words, capacity strengthening is a two-way street. Unlike the usual assumption of Northern researchers strengthening the capacity of Southern researchers, Northern researchers can learn not only about local context but also about different ways in which health in conflict is conceptualized and operationalized. That is, through the development and testing of metrics beyond death, injury and disease produced in the North to better assess the effects of wars and conflicts on health [2].

## Conclusion

In conclusion, we have demonstrated that there are several reasons for health-related local knowledge subjugation in the MENA region. The tools to address this issue are available but require long-term political commitment and will, paralleled with a contextually relevant research strategy and financial support. Such support will help local subjugated knowledge to resurface [3] so that the impact of these projects on the region’s health research, policy, and practice nexus becomes tangibly evident.
